# An Interesting Case in Practice

**Published:** 1900-10-15

**Authors:** G. A. Kennedy

**Affiliations:** Kent, Ohio


### AN INTERESTING CASE IN PRACTICE.

BY G. A. KENNEDY, D.D.S., KENT, OHIO.

There are two reasons why I wish to present to you the following case in practice. First, because it was one of interest and anxiety, and second, because it may possibly prove beneficial to some of our younger members.

Early last spring a man of thirty-five years presented himself to me with an alveolar abscess. The roots of an inferior right first molar was the cause of the irritation. It being night, and the roots not perceptible, instructions were given for him to return on the next day in order to have the offending members removed. However, he failed to appear until some weeks later, when he presented a much larger face than on the previous occasion. Having nothing but artificial light, and the roots still being invisible, the gum was lanced, in hopes that pus might be reached, but all to no purpose. Then a probe was used, and again no pus. The patient was instructed to place a split raisin over the part where the abscess was most likely to point, and the fact emphasized for the patient to return the next day. This he did, when the roots were removed, but not a drop of pus came with them. Pain had ceased and the patient was given a quantity of listerine, accompanied by the words that the climax of the trouble was reached, and that, no doubt, the abscess would point either through the socket where the roots were extracted, or else where the gum had been lanced. But such was not the case. On the following day the man returned with his face still paining him, and, of course, more swelling, involving the submaxillary gland. Upon being informed that pus must be reached, he said he could not endure the probing again and returned home to use the raisin once more.

After a few hours the family physician was summoned, who advised a poultice of hot flaxseed to be placed over the swelling. This was diligently pursued for several hours, when the physician desired me to call with him to see the case, the patient by this time not being able to come to the office. The swelling had extended well down on the neck, and it was with difficulty that the man could open his mouth. There was no sign of the pus pointing, and the hot poultices were continued. The swelling, however, increased, extending down to the clavicle, and up to the temple, while the tissues presented that board-like induration, so often seen in such cases.

The pharynx and tonsils were badly swollen, and deglutition was performed with difficulty. It was now thought best to make an incision upon the face, where pus seemed nearest the surface. Again it was a failure, so on the next day the patient was taken to Cleveland to be operated upon. His temperature by this time was 102°, and it was deemed best to proceed with the operation at once. Chloroform was administered. An incision was made on the face parallel with the body of the inferior maxillary. Then by Hilton's method of opening an abscess, the long-looked-for pus was found, underneath the deep fasia. It was of a thick, creamy consistency, with much odor. After removing as much of the pus as possible, and cleansing with bichloride, iodoform gauze was packed in the opening and the patient was dismissed. The wound was then dressed every other day with peroxide of hydrogen and a 2 percent solution of formaldehyde until the patient showed signs of recovery when the dressing was less frequent. It was fully a month, however, before the patient was able to return to work.

Now to what point are we, as dentists, expected to treat such cases? Was not the case just reported in its last stages, outside the sphere of dentistry, unless, however, one is making a specialty of oral surgery? At first there was merely an alveolar abcess, but at the last there was a typical case of "Ludwig's Angina," the prognosis of which is often grave. Out of fifty-eight cases reported and tabulated in literature, there was a mortality of 57 percent.

In some cases pus was reached, while in others none was to be found. At times there will be intense pressure and swelling of the side of the face and neck, resulting from the roots of teeth, but without a drop of pus being present. This is how we are to tell whether or not a deep cervical abscess is forming: If pus is accumulating there will usually be a rising temperature, though not always. You will find, by carefully passing the top of your finger over the swollen part, a soft area. This area will present a dusky, reddish appearance, at other times there will be intense swelling from the chin down to the clavicle, showing that board-like "infiltration" so often seen. The treatment of these cases should be as follows: A tonic and cathartic should be administered. In the early stages of the swelling, poultices or hot fomentations ought to be applied until either the swelling subsides, or else there is a strong evidence of pus. When one is convinced that pus is forming, the case should immediately be turned over to a surgeon. The dentist ought no longer to retain the case when developments have reached this stage. It has gone beyond an aveolar abscess with extended inflammation, and has grown into a deep cervical abscess which is by far more serious. At this juncture no time should be lost in evacuating the pus. Even now there is danger of gangrene and edema of the larynx, which may necessitate the performance of tracheotomy. In severe cases retropharnyngeal abscesses have been known to form and then the pus may point into the pharynx or treachea, in such a quantity as to cause asphyxia. A number of serious complications may result from these deep-seated abscesses.

However, they are serious enough, and I believe it is our duty as dentists to remove every old root that is useless, even though at the time it may be causing no trouble. And in case we have an aveolar abscess, to remove that pus if such a thing be possible.—*Ohio Dental Journal.*

